# Pre-pregnancy and early pregnancy dietary behavior in relation to maternal and newborn health in the Norwegian Fit for Delivery study – a post hoc observational analysis

**DOI:** 10.29219/fnr.v62.1273

**Published:** 2018-08-08

**Authors:** Elisabet R. Hillesund, Elling Bere, Linda R. Sagedal, Ingvild Vistad, Hilde L. Seiler, Monica K. Torstveit, Nina C. Øverby

**Affiliations:** 1 Department of Public Health, Sports and Nutrition, University of Agder, Kristiansand, Norway; 2 Department of Obstetrics and Gynecology, Sørlandet Hospital HF, Kristiansand, Norway; 3 Department of Research, Sørlandet Hospital HF, Kristiansand, Norway

**Keywords:** diet, neonatal outcome, diet score, preconception, preconception diet, pregnancy complications, pregnancy health, preeclampsia, preterm birth, gestational weight gain

## Abstract

**Background:**

Randomized controlled trials targeting maternal dietary and physical activity behaviors during pregnancy have generally failed to accomplish reductions in the prevalence of adverse maternal and neonatal outcomes. Interventions carried out during pregnancy could thus be missing the mark in maximizing intervention health benefit.

**Objective:**

To investigate whether *pre-pregnancy* and *early pregnancy* dietary behavior as reported at inclusion into the Norwegian Fit for Delivery (NFFD) trial was associated with maternal and neonatal outcomes irrespective of subsequent randomization assignment.

**Design:**

The study is a post-hoc observational analysis of data from a randomized controlled lifestyle intervention. We constructed two diet scores from participant responses to a 43-item questionnaire that addressed dietary behavior in retrospect (*pre-pregnancy* diet score) and dietary behavior at inclusion (*early pregnancy* diet score), respectively. The diet scores ranged from 0 to 10, with higher score reflecting healthier dietary behavior. Associations between diet scores and maternal and neonatal health outcomes were estimated in multivariate logistic regression models.

**Results:**

A total of 591 women were eligible for analysis. A one-point increase in *pre-pregnancy* diet score was associated with lower odds of excessive gestational weight gain (GWG) (odds ratio [OR]_adj_: 0.92; 95% confidence interval [CI]: 0.84–1.00, *p* = 0.050), preterm delivery (OR_adj_: 0.81; 95% CI: 0.68–0.97, *p* = 0.019), and birthweight ≥ 4,000 g (OR_adj_: 0.88; 95% CI: 0.78–0.99, *p* = 0.038). A one-point increase in *early pregnancy* diet score was associated with lower odds of excessive GWG (OR_adj_: 0.88; 95% CI: 0.79–0.97, *p* = 0.009), preterm delivery (OR_adj_: 0.82; 95% CI: 0.67–0.99, *p* = 0.038), and preeclampsia (OR_adj_: 0.78; 95% CI: 0.62–0.99, *p* = 0.038).

**Discussion:**

Higher diet score either pre-pregnancy or in early pregnancy was protectively associated with excessive GWG and preterm delivery, whereas the protective association with high birthweight was confined to *pre-pregnancy* diet and with preeclampsia to *early pregnancy* diet.

**Conclusions:**

Both pre-pregnancy and early pregnancy dietary behavior was associated with important maternal and neonatal health outcomes in the NFFD dataset.

Excessive gestational weight gain (GWG) has been identified as a potentially modifiable risk factor for adverse pregnancy outcomes based on consistent findings from several observational studies (1). In the randomized controlled Norwegian Fit for Delivery (NFFD) study, we targeted maternal weight gain through a lifestyle intervention during pregnancy and demonstrated that dietary advice and supervised exercise groups during pregnancy improved dietary behavior and physical activity level, and resulted in lower GWG compared to the control group who received standard care (2). There was, however, no accompanying reduction in common pregnancy complications such as gestational diabetes mellitus (GDM), preeclampsia, or preterm delivery, and no significant between-group differences in birthweight or other neonatal outcomes following the intervention. Other large and well-designed randomized controlled trials (RCTs) have reported similar findings, namely, a modest effect of pregnancy interventions on diet quality, physical activity level, and reduced GWG, but little or no effect on other measurable aspects of maternal and neonatal health (3–5). An individual patient data meta-analysis compiling data from several RCTs targeting lifestyle during pregnancy recently confirmed the lack of intervention effect on pregnancy complications and neonatal health outcomes despite self-reported improvement in diet and physical activity (6). Findings from large prospective observational studies have, on the other contrary, repeatedly indicated protective associations between healthier maternal diet and pregnancy complications such as preeclampsia (7, 8), preterm delivery (8–12), and GDM (13).

These seemingly contradictory findings from observational versus experimental studies could possibly be reconciled by taking into account the time window represented by the dietary data. The abovementioned observational studies information on maternal diet was collected during pregnancy. High correlations have previously been demonstrated between presently reported diet and diet reported several years ahead in the general population (14). Dietary data collected during pregnancy may thus simultaneously represent longer-term diet and complicate the interpretation of whether observed associations between diet and pregnancy outcomes relate to diet during pregnancy per se or rather to maternal pre-pregnancy diet and nutritional status before conception. This distinction could be of considerable public health interest, because pregnancy complications such as GDM, preterm birth, and preeclampsia are relatively common and may hamper maternal and newborn immediate and long-term health with associated large individual and socioeconomic life course costs (15). Effective prevention strategies and relevant stages for prevention are therefore being searched for (16).

In the NFFD study, participants responded to questions about diet and dietary behavior both at the time of inclusion around week 15 of pregnancy and, in retrospect, to identical questions covering the period before getting pregnant. This left us with the opportunity to investigate both pre-pregnancy and early pregnancy dietary behavior in relation to subsequent maternal and neonatal outcomes and compare the respective effect sizes. The aim of this observational post-hoc analysis was thus to investigate whether NFFD participants’ dietary behavior *before* inclusion, assessed both pre-pregnancy and in early pregnancy, was associated with aspects of maternal and neonatal health irrespective of subsequent randomization assignment.

## Methods

The present paper is a post-hoc analysis carried out among pregnant women participating in the NFFD study (17). We treated the study population as a cohort for investigating pre-intervention diet–outcome associations independently of randomization assignment but took potential randomization-related effects into account by adjusting for randomization status in the analyses and by carrying out sensitivity analyses confined to the control group. Although the main study and effects of the lifestyle intervention have been thoroughly described elsewhere (2), they have been briefly described below.

### The Norwegian Fit for Delivery trial

The NFFD study is a population-based lifestyle RCT carried out among nulliparous pregnant women in Southern Norway between 2009 and 2013 (ClinicalTrials.gov ID NCT01001689). The main aim of the study was to facilitate optimal GWG through dietary advice and twice-weekly supervised exercise lessons, and thereby reducing the number of infants with high birthweight. The study protocol and the effectiveness of the intervention on various outcomes have been published previously (17–21).

### Participants

Women were eligible for participation if they were 18 years or older, nulliparous, had pre-pregnancy body mass index (BMI) ≥19 kg/m^2^, were 20 weeks pregnant or less at inclusion, carrying a single fetus, literate in Norwegian or English, and had provided written consent (2). Exclusion criteria comprised pre-existing diabetes mellitus, disabilities precluding participation in a physical fitness program, ongoing substance abuse, or planned relocation outside the study area before delivery. Out of 1,610 potentially eligible women, a total of 606 nulliparous women agreed to participate and were consecutively enrolled from eight health care clinics between September 2009 and February 2013. Mean gestational age at inclusion was 15 weeks. Participants were randomly assigned to the control (*n* = 303) or intervention group (*n* = 303), respectively. Twelve women were later withdrawn from the study because of miscarriage (*n* = 6), twin pregnancy (*n* = 2), and relocation outside study area (*n* = 4). One woman was excluded from participation in the trial due to very low BMI after being mistakenly included, and two women withdrew from the trial without giving permission to use data. A total of 591 participants were thus included in intention-to-treat analyses in the main study and were also eligible for the observational analyses in the present study.

### The 10 dietary behaviors that were targeted in the diet intervention

The diet intervention aimed at facilitating optimal GWG and otherwise promoting a healthy pregnancy through simple diet rules aimed at heightening participant reflection on dietary behavior (see below). We based the intervention components on 10 dietary recommendations developed specifically for the study. The rationale for the 10 recommendations has been published previously (22).

The 10 dietary recommendations in the NFFD trial are the following (19):

1. Eat regular meals
2. Drink water when thirsty
3. Eat vegetables with dinner every day
4. In-between meals – choose fruits and vegetables
5. Eat sweets and snacks only when you really appreciate it
6. Choose small portion sizes of unhealthy foods
7. Limit your intake of added sugar
8. Limit your intake of salt
9. Do not eat beyond satiety
10. Read nutritional labels

Women randomized to the intervention group received an illustrated booklet describing the dietary recommendations and their simplified rationale shortly after randomization. The recommendations were reinforced and tailored to the individual in two telephone sessions with a trained advisor, scheduled approximately 4–6 weeks apart. The intervention also comprised access to supervised exercise classes twice weekly, including strength training and cardiovascular exercise at moderate intensity. Women in the control group received routine pregnancy care but answered the same questionnaires and received the same medical follow-up as intervention participants, including extra ultrasound investigations and blood tests. The present paper addresses pre-intervention dietary behavior in relation to maternal and neonatal health outcomes.

### The Norwegian Fit for Delivery diet score

We operationalized pre-intervention dietary behavior as a *pre-pregnancy* and an *early pregnancy* diet score, respectively. The scores were designed to reflect participant degree of compliance with the 10 dietary recommendations prior to inclusion into the study. Both diet scores were built from 10 subscales, each subscale referring to a corresponding dietary behavior. The subscales were built from participants’ responses to the baseline questionnaire that included a 43-item questionnaire with food frequency questions (FFQ) and questions about aspects of dietary behavior. All 43 questions addressed pre-pregnancy diet in retrospect, and present diet at the time of inclusion (e.g. ‘how often did you drink water before you became pregnant?’ and ‘how often do you drink water now?’). The questionnaire only covered selected aspects of diet and dietary behavior, mainly the dietary aspects that were targeted in the NFFD intervention (22). The subscales could be single variables or sum scores constructed from relevant questionnaire responses. Each subscale was dichotomized with the sample median as cutoff, and participants with the healthier behavior were assigned ‘1’ in each subscale, whereas the other half of the sample was assigned ‘0’. Individual diet scoring thus ranged from 0 to 10, with higher score indicating healthier behavior. For some of the analyses, participants were categorized as having low [0–3], medium [4–5], or high [6–10] diet score. Supplementary Table 1 provides an overview of the variables that were included in each subscale including cutoffs for scoring in each individual subscale. In brief, the behaviors that yielded scoring in the pre-pregnancy and early pregnancy subscales were as follows:

1. having ≥24 main meals/week (≥25 in the early pregnancy subscale)
2. 44% or more of drinking events being water (≥46% in the early pregnancy subscale)
3. having vegetables with dinner ≥5 times/week
4. choosing fruits or vegetables for in-between meal snacks ≥3 times/week (≥5 times/week in the early pregnancy subscale)
5. never eating sweets and snacks without appreciation
6. buying small portion size of one or more unhealthy food items (soda, salty crisps, or chocolate)
7. consuming sugar-rich food items once a day or less
8. consuming fast-foods, snacks, or other salty food less than daily
9. eating beyond satiety less than once a week
10. reading nutrition labels on foods sometimes or often.

A detailed description of the construction of the diet score and its test–retest reliability has been published previously (22).

### Maternal and child outcomes

Outcomes in the present study were excessive and inadequate GWG, preeclampsia, preterm delivery, GDM, and various measures of birthweight. Birth records and hospital charts were reviewed in retrospect to validate information on maternal and neonatal outcomes (2)

All participants were weighed at their healthcare clinic at inclusion, and at Sorlandet Hospital at 30 gestational-weeks (Tanita BC 418, Tokyo, Japan). Height was measured with a stadiometer (Seca Leicester, Hamburg, Germany). Pre-pregnancy BMI (kg/m^2^) was calculated based on self-reported pre-pregnancy weight (kg) and measured height (m). Participants were also weighed on admission to the delivery ward. If admission weight was not available, the last weight in the prenatal record was recorded with its corresponding date. Total weight gain was calculated for women who delivered at ≥37 gestational-weeks with measured weight available within 2 weeks of admission (2). Excessive GWG was defined as pregnancy weight gain measured at term exceeding the optimal range proposed by the 2009 Institute of Medicine (IOM) guidelines, that is, >16.0 kg if normal weight, >11.5 kg if overweight, and >9.0 kg if obese pre-pregnancy (1). Inadequate GWG was defined as weight gain below the BMI-specific optimal range, that is, <11.5 kg if normal weight, <7.0 kg if overweight, and <5.0 kg if obese pre-pregnancy (1). Inadequate and excessive GWG were treated as dichotomous variables in the analyses (excessive GWG yes/no and inadequate GWG yes/no).

Participants underwent a glucose tolerance test in gestational week 30, with measurement of fasting serum glucose after overnight fasting, and postprandial level 2 h after intake of 75 g of glucose. Glucose levels ≥7.8 mmol/l at 2 h were classified as elevated, based on 2006 WHO criteria (23). The diagnosis of GDM was subsequently ascertained from hospital charts.

Preeclampsia was diagnosed based on guidelines adopted by the Norwegian Federation of Obstetricians and Gynecologists; an increase in blood pressure to at least ≥140 systolic or 90 mm Hg diastolic after 20th gestational week combined with proteinuria (protein excretion of at least 0.3 g/24 h or ≥1+ on dip-stick), both measured at least twice (2, 24). Severe preeclampsia was defined as preeclampsia before 34 weeks of pregnancy and/or severity of symptoms, as documented in hospital charts. Cases of eclampsia and HELLP-syndrome (preeclampsia affecting hemolysis, liver function, and platelet counts) were included as severe preeclampsia cases.

Preterm delivery was defined as delivery before 37 completed weeks of gestation. Estimated date of confinement was determined as part of routine prenatal care for all participants, based on ultrasound examinations supplemented with date of the last menstrual cycle.

We assessed birthweight according to widely used cutoffs for macrosomia (≥4,000 g and ≥4,500 g) and low birthweight (<2,500 g) (25), and relative to national reference values for gestational age and gender. Being small for gestational age (SGA) was defined as birthweight below the 10th percentile and being large for gestational age (LGA) was equivalent to birthweight ≥90th percentile, both calculated according to sex and gestational age-specific references from the Medical Birth Registry of Norway (MBRN) (26).

### Sociodemographic variables and potential confounders

Information on maternal age, education, marital status, occupation, income, smoking, pre-pregnancy weight and early pregnancy physical activity level was collected from the baseline questionnaire completed upon inclusion. BMI was calculated from self-reported pre-pregnancy weight and height measured with a stadiometer at inclusion (Secca Leicester, Hamburg, Germany). Physical activity level was assessed with the International Physical Activity Questionnaire short form (IPAQ-SF) (27, 28) that quantifies frequency and duration of physical activity in the intensity categories: vigorous, moderate, walking and sitting during the last 7 days (27). In addition to intensity, frequency and duration of physical activity are assessed. Responses were scored according to IPAQ-SF analysis algorithms into three categories denoting low, medium or high physical activity level.

### Statistics

Statistical analyses were performed with SPSS for IBM statistical software package version 24.0 (IBM Corporation, Armonk, NY, USA). A two-sided *p*-value of ≤ 0.05 was considered significant. Maternal age, weight, height, and BMI are presented with mean and standard deviation (SD). Sociodemographic variables are presented with number and proportions (%). We calculated mean diet score for each category of the sociodemographic variables to visualize covariance between *pre-pregnancy* and *early pregnancy* diet scores and potential confounders.

We compared dietary intake across diet score categories and presented this information with median and interquartile range (IQR) as Supplementary Table 2. Prevalence of maternal and neonatal outcomes were similarly compared across low, medium and high diet score categories and tested for trend across categories with the Mantel-Haenszel statistics (29).

We estimated crude and adjusted odds ratios (OR) and 95% confidence intervals (CI) for all outcomes with the continuous *pre-pregnancy* and *early pregnancy* diet scores as main exposure in separate tables. In the multivariate models, we included the following potential confounders: maternal age at inclusion (continuous), marital status (husband/boyfriend/partner or living alone), pre-pregnancy BMI (continuous), educational attainment (≤12, 13–15, and ≥16 years), household income (≤400,000, 401,000–700,000, and >700,000 NOK/year, equivalent to <52,000, 52,000–91,000, and <91,000 dollars/year, assessed from exchange rate on 18 September 2017), and randomization assignment (control/intervention). In the *early pregnancy* analyses, we additionally included current smoking (yes/no). A total of 110 participants had missing information on early pregnancy physical activity level. We therefore fit a third *early pregnancy* model that included early pregnancy physical activity level (low, medium, or high) along with the other potential confounders.

Given the randomized controlled design, and the fact that the intervention group improved diet and physical activity behaviors compared to the control group between randomization and delivery (19), we performed sensitivity analyses by rerunning all pre-pregnancy and early pregnancy models confined to the control group who received no intervention.

### Ethics

Written informed consent was obtained from all participants before inclusion into the study. The study was approved by the Norwegian Regional Committee for Medical Research Ethics South East C (REK reference 2009/429). The authors assert that all procedures contributing to this work comply with the ethical standards of the Norwegian Regional Committee for Medical Research Ethics and with the Helsinki Declaration of 1975, as revised in 2008. The NFFD trial has the Clinical Trials registration: clinicaltrial.gov NCT0100168.

### Results

Descriptive information about the 591 participants is presented in Table 1. Mean age at inclusion was 28.0 years (SD 4.4, range18–44). Included women were 168.7 cm tall (SD 6.2) and weighed 67.5 kg (SD12.2). Mean pre-pregnancy BMI was 23.7 kg/m^2^ (SD 3.9). Mean *pre-pregnancy* and *early pregnancy* diet score across categories of the sociodemographic variables are presented in Table 1. There was a positive correlation with educational attainment for both diet scores (*p* < 0.001) and a negative correlation with smoking status (*p* < 0.05). Pre-pregnancy diet score was positively correlated with early pregnancy physical activity level (*p* = 0.029). Neither age, pre-pregnancy BMI, marital status, income nor occupation was significantly associated with the diet scores. A comparison of the dietary characteristics associated with low, medium, and high diet scores in both *pre-pregnancy* and in *early pregnancy* are presented in Supplementary Table 2. Higher diet score implied more frequent consumption of main meals, fruits, vegetables, and water, and less frequent consumption of sweetened beverages, sweets, and snacks. There was considerable correlation between the continuous *pre-pregnancy* and *early pregnancy* diet scores (r_Pearson_ = 0.59, *p* < 0.001).

**Table 1:** Maternal characteristics and correlation with pre-pregnancy and early pregnancy diet scores in the Norwegian Fit for Delivery study (*n* = 591)

Maternal characteristics	Number included	Percentage	Pre-pregnancy diet score Mean (SD)	Early pregnancy diet score Mean (SD)
**Age at inclusion (years)**	591			
** <25**	149	25.2	4.3 (2.1)	4.7 (1.9)
** 25–29**	273	46.2	4.5 (2.2)	4.9 (2.1)
** 30–34**	129	21.8	4.8 (2.1)	5.2 (2.1)
** 35+**	40	6.8	4.7 (2.4)	5.4 (1.9)
**Education (years)**	588			
** ≤12**	187	31.8	4.0 (2.1)	4.6 (2.0)
** 13–15**	192	32.7	4.6 (2.2)	4.9 (2.1)
** ≥16**	209	35.5	5.1 (2.1)	5.4 (2.0)
** Missing**	3			
**BMI category pre-pregnancy**	590			
** <25.0**	426	71.5	5.4 (1.9)	5.0 (1.6)
** 25.0–29.9**	119	20.8	4.4 (2.1)	4.8 (2.0)
** ≥30.0**	45	7.6	4.4 (2.4)	5.1 (1.9)
** Missing**	1			
**Current smoking**				
** No smoking**	589	96.1	4.6 (2.2)	5.0 (2.1)
** Current smoking**	23	3.9	3.1 (1.8)	4.1 (1.9)
** Missing**	2			
**Marital status**	589			
** Married/boyfriend/partner**	567	96.3	4.6 (2.2)	5.0 (2.1)
** Other**	22	3.7	4.2 (2.1)	4.5 (1.7)
** Missing**	2			
**Occupation**				
** Work outside home**	496	84.2	4.6 (2.2)	4.9 (2.0)
** Student**	51	8.7	5.1 (2.2)	5.5 (2.2)
** Unemployed**	23	3.9	4.2 (1.6)	4.8 (2.0)
** Sick leave/disabled**	11	1.9	3.5 (2.0)	4.9 (1.9)
** Homemaker**	8	1.4	3.6 (1.7)	4.1 (1.4)
** Missing**	2			
**Income (NOK)**				
** ≤400,000**	183	31.2	4.3 (2.2)	4.7 (2.0)
** 400,001–700,000**	163	27.8	4.5 (2.1)	5.1 (2.1)
** >700,000**	202	34.4	4.9 (2.2)	5.1 (2.0)
** Refrain from response**	39	6.6	4.2 (2.4)	4.9 (2.1)
** Missing**	4			
**Physical activity level in early pregnancy^a^**	481			
** Low activity**	127	26.4	4.2 (1.9)	4.7 (1.8)
** Medium activity**	280	58.2	4.8 (2.3)	5.3 (2.2)
** High activity**	74	15.4	5.0 (2.3)	5.0 (2.0)
** Missing**	110			
**Randomization status**	591			
** Control**	295	49.9	4.6 (2.2)	5.0 (2.1)
** Intervention**	296	50.1	4.5 (2.1)	5.0 (2.1)

BMI, Body Mass Index; NOK, Norwegian currency (1 US Dollar).

a Based on responses to the International Physical Activity Questionnaire short form (IPAQ-SF) and scored and categorized according to IPAQ analysis algorithms into physical activity categories (27)

### Maternal and newborn outcomes

Differences in prevalence of maternal and child outcomes with low, medium, and high *pre-pregnancy* and *early pregnancy* diet scores, respectively, are presented in Table 2. There were significant trends toward lower prevalence of excessive GWG, preterm delivery, and macrosomia, across *pre-pregnancy* diet score categories, but concurrent higher prevalence of SGA with higher diet scores. For the *early pregnancy* diet score, we observed significant trends toward lower prevalence of preterm delivery and macrosomia with higher diet score, and a concurrent higher prevalence of SGA.

**Table 2:** Difference in prevalence of maternal and neonatal outcomes across *pre-pregnancy* and *early pregnancy* diet score categories (*n* = 591)

Obstetrical outcomes	Included in the analysis	Number of cases	Pre-pregnancy diet score categories				Early pregnancy diet score categories			
	
			Low *N* = 195 (33.0%)	Medium *N* = 204 (34.5%)	High *N* = 192 (32.5%)	*p*-trend*	Low *N* = 151 (25.5%)	Medium *N* = 204 (34.5%)	High *N* = 236 (*n* = 39.9%)	*p*-trend*
**Adequacy of pregnancy weight gain (term)^a^**			%	%	%		%	%	%	
** Excessive**	531	258	52.7	49.2	42.8	**0.033**	49.7	51.3	44.8	0.197
** Inadequate**	527	110	19.4	22.8	20.8	0.546	19.6	22.3	20.7	0.604
**Gestational diabetes^b^**										
** Elevated 2-h glucose tolerance test (WHO criteria)**	582	53	6.3	13.9	6.9	0.808	5.4	12.5	8.6	0.432
**Preeclampsia^c^**										
** All cases combined**	582	25	4.7	3.5	4.8	0.965	7.4	2.5	3.9	0.151
** Severe preeclampsia/HELLP/eclampsia^d^**	582	15	3.1	2.0	2.7	0.772	5.4	1.0	2.1	0.090
**Preterm delivery**										
** Prior to 37 weeks (all)**	591	34	8.7	5.9	2.6	**0.010**	12.6	3.4	3.4	**<0.001**
** Prior to 37 weeks (preeclampsia excluded)**	557	24	7.1	4.6	1.1	**0.005**	9.4	3.1	2.2	**0.002**
**Neonatal outcomes**										
** Birthweight > 4,000 g (term)**	557	75	18.0	12.5	10.2	**0.029**	17.4	14.7	10.5	**0.041**
** Birthweight > 4,500 g (term)**	557	7	1.1	1.0	1.6	0.677	3.0	1.0	0.4	**0.042**
** LGA > 90th centile^e^**	591	18	3.1	4.4	2.6	0.390	4.0	3.9	1.7	0.168
** Birthweight <2.5 kg (term)**	557	7	1.1	1.6	1.1	0.958	0.0	2.0	1.3	0.385
** SGA < 10th centile^e^**	591	58	8.2	6.9	14.6	**0.036**	6.6	8.8	12.7	**0.043**
** Newborn birthweight, mean (SD)**	591		3,440 (524)	3,452 (523)	3,397 (490)	0.528^f^	3,412 (585)	3,476 (501)	3,403 (470)	0.291^f^

LGA, large for gestational age; SGA, small for gestational age.

* *P*-trend across diet score categories (Mantel-Haenszel statistics).

a Weight gain outside Institute of Medicine (IOM) 2009 BMI-specific recommendations, calculated for term pregnancies only (1).

b WHO 1999 criteria at gestational week 30: Elevated 2-h glucose ≥7.8 mmol/l (23).

c Based on guidelines adopted by the Norwegian Federation of Obstetricians and Gynecologists; an increase in blood pressure to at least ≥140 systolic or 90 mm Hg diastolic after 20th gestational week combined with proteinuria (protein excretion of at least 0.3 g/24 h or ≥1+ on dip-stick), both measured at least twice (24). Nine participants did not have information on preeclampsia recorded.

d Defined as preeclampsia diagnosed before 34 weeks of pregnancy and/or severity of symptoms, as documented in hospital charts. Cases of eclampsia and HELLP-syndrome were included.

e Birth weight centiles calculated according to offspring sex and gestational age, based on data from the Medical Birth Registry of Norway (MBRN) (26).

f One-way ANOVA.

Mean GWG was 15.0 kg (SD 6.0). The overall prevalence of excessive and inadequate GWG was 48.6 and 20.9%, respectively, leaving 30.5% with optimal GWG. There were significant inverse associations between a one-point increase in *pre-pregnancy* and *early pregnancy* diet scores and odds of excessive GWG in crude and adjusted models (Tables 3 and 4) but no association between the diet scores and inadequate GWG.

**Table 3:** Associations between *pre-pregnancy* NFFD diet score and maternal and newborn outcomes (*n* = 591)

Obstetrical outcomes	No. included in the analysis	No. of cases	Pre-pregnancy model 1 Crude			Pre-pregnancy model 2 Adjusted^a^		
			OR	95% CI	*p*-value	OR^a^	95% CI	*p*-value
**Adequacy of weight gain (at term)^b^**								
** Excessive**	528	256	**0.91**	**0.84–0.98**	**0.019**	**0.92**	**0.84–1.00**	**0.050**
** Inadequate**	524	110	1.03	0.93–1.13	0.576	1.02	0.92–1.12	0.766
**Gestational diabetes^c^**								
** Elevated 2-h glucose tolerance test (WHO criteria)**	578	53	1.06	0.93–1.20	0.403	1.07	0.94–1.23	0.314
**Preeclampsia^d^**								
** Preeclampsia total**	578	25	0.99	0.82–1.19	0.900	0.97	0.81–1.18	0.784
** Severe preeclampsia/HELLP/eclampsia^e^**	578	15	0.93	0.73–1.18	0.564	0.93	0.73–1.19	0.573
**Preterm delivery**								
** Prior to 37 weeks**	586	34	**0.81**	**0.68–0.96**	**0.014**	**0.81**	**0.68–0.97**	**0.019**
** Prior to 37 weeks (preeclampsia cases excluded)**	553	24	**0.76**	**0.62–0.93**	**0.008**	**0.77**	**0.62–0.95**	**0.013**
**Neonatal outcomes**								
** Birthweight > 4,000 g (term)**	552	75	**0.87**	**0.77–0.98**	**0.016**	**0.88**	**0.78–0.99**	**0.038**
** Birthweight > 4,500 g (term)**	552	7	0.82	0.57–1.17	0.275	0.76	0.52–1.14	0.183
** LGA >90th centile^f^**	591	18	0.80	0.63–1.00	0.049	0.91	0.64–1.02	0.071
** Birthweight < 2.5 kg (term)**	552	7	0.90	0.64–1.28	0.567	0.92	0.63–1.33	0.646
** SGA < 10th centile^f^**	586	57	1.08	0.95–1.22	0.242	1.09	0.95–1.24	0.217

LGA, large for gestational age; SGA, small for gestational age.

a Multivariable associations between pre-pregnancy diet score and outcomes are expressed as odds ratios (OR) with 95% confidence intervals (95% CI) and corresponding *p*-values, adjusted for maternal age (continuous), educational attainment (≤12, 13–15, ≥6 years), marital status (cohabiting yes/no), family income (4 categories), pre-pregnancy BMI (continuous), and randomization assignment (control/intervention).

b Weight gain outside Institute of Medicine (IOM) 2009 recommendations, calculated for term pregnancies only (1).

c WHO 1999 criteria at gestational week 30: Elevated 2-h glucose ≥7.8 mmol/l (23).

d Based on guidelines adopted by the Norwegian Federation of Obstetricians and Gynecologists; an increase in blood pressure to at least ≥140 systolic or 90 mm Hg diastolic after 20th gestational week combined with proteinuria (protein excretion of at least 0.3 g/24 h or ≥1+ on dip-stick), both measured at least twice (24).

e Defined as preeclampsia diagnosed before 34 weeks of pregnancy and/or severity of symptoms, as documented in hospital charts. Cases of eclampsia and HELLP-syndrome were included.

f Birth weight centile calculated according to offspring sex and gestational age, based on data from the Medical Birth Registry of Norway (MBRN) (26).

**Table 4:** Associations between *early pregnancy* NFFD diet score and maternal and newborn outcomes (*n* = 591)

Obstetrical outcomes	No. included in the analysis	No. of cases	Early pregnancy model 1 Crude			Early pregnancy model 2 Adjusted^a^			Early pregnancy model 3 Adjusted^b^		
			OR	95% CI	*p*-value	OR^a^	95% CI	*p*-value	OR^b^	95% CI	*p*-value
**Adequacy of weight gain (term)^c^**											
** Excessive**	528	256	**0.90**	**0.83–0.98**	**0.019**	**0.90**	**0.83–0.99**	**0.024**	**0.88**	**0.79–0.97**	**0.009**
** Inadequate**	524	110	1.05	0.95–1.17	0.331	1.05	0.94–1.17	0.381	1.02	0.95–1.19	0.374
**Gestational diabetes^d^**											
** Elevated 2-h glucose tolerance test (WHO criteria)**	578	53	1.02	0.88–1.17	0.815	1.00	0.86–1.15	0.949	0.98	0.83–1.16	0.810
**Preeclampsia^e^**											
** Preeclampsia total**	578	25	0.93	0.76–1.13	0.461	0.90	0.73–1.10	0.325	**0.78**	**0.62–0.99**	**0.038**
** Severe preeclampsia/HELLP/eclampsia^f^**	582	15	0.91	0.71–1.17	0.457	0.90	0.67–1.17	0.429	0.74	0.54–1.01	0.060
**Preterm delivery**											
** Prior to 37 weeks**	587	34	**0.77**	**0.65–0.92**	**0.004**	**0.77**	**0.64–0.92**	**0.005**	**0.82**	**0.67–0.99**	**0.038**
** Prior to 37 weeks (preeclampsia cases excluded)**	555	24	**0.77**	**0.62–0.95**	**0.014**	**0.77**	**0.62–0.96**	**0.018**	0.81	0.64–1.02	0.068
**Neonatal outcomes**											
** Birthweight > 4,000 g (term)**	552	75	**0.86**	**0.76–0.98**	**0.018**	0.89	0.78–1.01	0.061	0.91	0.79–1.05	0.185
** Birthweight > 4,500 g (term)**	552	7	**0.58**	**0.39–0.89**	**0.011**	**0.54**	**0.35–0.84**	**0.006**	0.60	0.33–1.10	0.097
** LGA >90th centile^g^**	586	18	0.80	0.63–1.01	0.060	0.79	0.62–1.01	0.062	0.80	0.60–1.09	0.166
** Birthweight < 2.5 kg (term)**	552	7	1.18	0.82–1.71	0.368	1.25	0.84–1.86	0.267	1.20	0.79–1.83	0.387
** SGA < 10th centile^g^**	586	57	1.12	0.98–1.28	0.107	1.13	0.98–1.29	0.089	1.10	0.94–1.28	0.225

LGA, large for gestational age; SGA, small for gestational age.

a Multivariable associations between early pregnancy diet score and the outcomes are expressed as odds ratios (OR) with 95% confidence intervals (95% CI) and corresponding *p*-values. A *p*-value of ≤ 0.05 is considered significant. Early pregnancy model 2 is adjusted for maternal age (continuous), educational attainment (≤12, 13–15, ≥6 years), marital status (married/cohabiting yes/no), family income (4 categories), pre-pregnancy BMI (continuous), current smoking (yes/no), and randomization assignment (control/intervention).

b Model 3 is adjusted for all variables in model 2 and in addition early pregnancy physical activity level (3 categories). There was a large number of missing values in model 3 due to missing information on early pregnancy physical activity level (*n* = 110).

c Weight gain outside Institute of Medicine (IOM) 2009 recommendations (1)

d WHO 1999 criteria at gestational week 30: Elevated 2-h glucose ≥7.8 mmol/l (23).

e Based on guidelines adopted by the Norwegian Federation of Obstetricians and Gynecologists; an increase in blood pressure to at least ≥140 systolic or 90 mm Hg diastolic after 20th gestational week combined with proteinuria (protein excretion of at least 0.3 g/24 h or ≥1+ on dip-stick), both measured at least twice (24).

f Defined as preeclampsia diagnosed before 34 weeks of pregnancy and/or severity of symptoms, as documented in hospital charts. Cases of eclampsia and HELLP-syndrome were included.

g Birth weight percentile calculated according to offspring sex and gestational age, based on data from the Medical Birth Registry of Norway (MBRN) (26).

A total of 53/582 (9.1%) NFFD participants were diagnosed with GDM. The highest prevalence of GDM was observed among women in the medium diet score category, whether assessed *pre-pregnancy* (13.9%) or in *early pregnancy* (12.5%) (Table 2). There was, however, no association between *pre-pregnancy* and *early pregnancy* diet scores and GDM in multivariate analyses (Tables 3 and 4).

A total of 25/582 participants (4.3%) developed preeclampsia, with 15/582 (2.6%) classified as severe cases. Early pregnancy diet score was protectively associated with preeclampsia risk in model 3 when adjusted for pre-pregnancy physical activity level in addition to other potential confounders (Table 4). The same trend was observed for severe preeclampsia, although not significant.

A total of 34/591 women (5.8%) delivered preterm. Women with low *early pregnancy* diet score had the highest prevalence of preterm delivery (12.5%), while women with high diet score *pre-pregnancy* had the lowest prevalence (2.6%) (Table 2). There were significant inverse associations between the diet scores and preterm delivery, whether assessed pre-pregnancy or in early pregnancy (Tables 3 and 4). The same pattern was observed when pregnancies complicated by preeclampsia were excluded from the analysis.

A total of 75/557 (13.5%) newborns had birthweight ≥4,000 g at term, and 7/557 (1.3%) had birthweight ≥4,500 g. A total of 18/591 (3.0%) were classified as LGA. Both the *pre-pregnancy* and *early pregnancy* diet score were inversely associated with birthweight above 4 kg in crude models, but the association was attenuated and no longer significant in the adjusted models (Tables 3 and 4). The *early pregnancy* diet score was associated with lower risk of birthweight above 4.5 kg, but the association was attenuated after adjustment for *early pregnancy* physical activity level (Table 4). There were no significant associations between the diet scores and other measures of fetal growth.

### Sensitivity analyses

There was no formal interaction between randomization assignment and the diet scores. We nevertheless reran all *pre-pregnancy* and *early pregnancy* models confined to the control group to assess the associations in a non-intervention setting. This made no substantial difference in the direction or magnitude of the estimates although some of the associations were no longer significant because of the smaller sample (Supplementary Tables 3a and 3b).

### Discussion

In this study, we investigated whether and how degree of maternal compliance with a set of predefined dietary behaviors assessed both pre-pregnancy and in early pregnancy was associated with maternal and neonatal outcomes in the NFFD dataset. For the outcomes excessive GWG and preterm delivery, respectively, we found protective associations of similar magnitude with *pre-pregnancy* and *early pregnancy* dietary behavior. Lower odds of high birthweight were only observed with increasing *pre-pregnancy* diet score, whereas lower odds of preeclampsia were only observed with increasing *early pregnancy* diet score. No association between the two diet scores and GDM was observed in this sample.

To our knowledge, no other study has investigated both pre-pregnancy and early pregnancy dietary behavior in the same individuals in relation to subsequent maternal and neonatal outcomes. Having done so, allows for speculation regarding sensitive periods for the role of diet and dietary behaviors in the prevention of adverse pregnancy outcomes. Our findings suggest a protective influence of healthier pre-pregnancy dietary behavior on GWG, preterm delivery, and newborn macrosomia, but possibly also an independent protective influence of early pregnancy dietary behavior on preeclampsia risk. Although aspects of dietary behavior changed from pre-pregnancy to early pregnancy among NFFD participants (30, 31), the correlation between the two diet scores in the present study was high.

### Gestational weight gain

The protective associations observed between pre-pregnancy and early pregnancy dietary behavior and excessive GWG support the relevance of targeting the chosen dietary behaviors for optimizing GWG. A recent observational study from Greece reported that higher adherence to the Mediterranean diet prior to pregnancy was associated with decreased risk for deviation from the maternal recommended GWG (32).

NFFD women with high diet score *pre-pregnancy* had similar prevalence of excessive GWG as women in the intervention group (41.6% vs. 42.8%) (2) and almost one in two participants in the NFFD cohort exceeded BMI-specific recommendations for GWG which compares well with findings from the MoBa cohort (33). Given the high prevalence of excessive GWG in all diet score categories, more research is needed to identify successful interventions for optimizing GWG.

### Preterm delivery

Preterm birth is strongly associated with perinatal and infant mortality (34), and even late preterm birth may have negative consequences for long-term health and development (35). Rates of preterm birth vary widely among and within countries, and modifiable determinants are searched for (16). We observed inverse associations of similar strength between *pre-pregnancy* and *early* pregnancy diet scores and subsequent preterm delivery. Our findings are in agreement with previous observations of protective associations between healthy dietary patterns during pregnancy and subsequent preterm delivery (8, 9, 11, 12), and higher risk with unhealthy dietary patterns (12, 36). In the National Danish Birth Cohort (DNBC), a consistent dose–response association between Western diet score and risk of preterm delivery was observed (OR: 1.30, 95% CI: 1.13, 1.49 for highest vs. lowest quintile). Grieger et al. derived dietary patterns from retrospectively reported *pre-pregnancy* diet in 309 women and found that higher pre-pregnancy score on a high-protein/fruit pattern was associated with lower odds of subsequent preterm delivery (OR: 0.31; 95% CI: 0.50, 0.91 for each SD higher score) (37). The NFFD diet score reflect constellations of maternal dietary behaviors with potential favorable impact on maternal hormones, metabolism, immunologic factors, inflammation, antioxidant defense, and energy balance, all of which might influence the risk of preterm delivery (34). Given the magnitude and strength of the associations between *pre-pregnancy* and *early pregnancy* diet score and preterm delivery, it was somewhat surprising that there was no reduction in preterm delivery in the NFFD intervention group. If a true relationship exists between pregnancy dietary behavior and risk of preterm delivery, this lack of intervention effect on preterm delivery could imply a time-dependent association, that is, that interventions need to be implemented earlier in pregnancy or even before pregnancy to be effective. Other possibilities are insufficient intensity of the NFFD intervention to impact on preterm delivery risk, insufficient dietary difference between control and intervention group, or that other aspects of the lifestyle intervention negated potential protective effects of diet.

### Preeclampsia

Preeclampsia is another serious complication of pregnancy that may threaten maternal survival and severely affect fetal growth and development (38). There was no association between *pre-pregnancy* diet score and preeclampsia in the present study. A significant protective association was, however, observed between *early pregnancy* dietary behavior when the model was adjusted for physical activity level (Table 4), indicating that the protective association with early pregnancy diet was negatively confounded by physical activity level. Fewer preeclampsia cases occurred in the intervention group, but the difference was not significant (OR: 0.65; 95% CI: 0.29–1.47) (2). Importantly, the NFFD trial was not powered to detect between-group differences for this outcome. A meta-analysis of lifestyle interventions during pregnancy by Allen et al. found that dietary interventions resulted in an estimated 33% reduction in preeclampsia prevalence (39), supporting an independent influence of dietary factors during pregnancy. We only found one other study that prospectively investigated associations between *pre-pregnancy* diet and subsequent hypertensive disorders of pregnancy. This study reported a protective dose–response association between *pre-pregnancy* consumption of a Mediterranean-style dietary pattern and subsequent risk of hypertensive disorders of pregnancy (quartile 4 vs. quartile 1: RR, 0.58; 95% CI, 0.42, 0.81) (40).

### Newborn birthweight

Only seven children had birthweight ≥4,500 g, so associations between the diet scores and this outcome should be interpreted with caution. The observation that higher diet score was associated with lower odds of high birthweight might point to pre-pregnancy and the first trimester as a window of opportunity regarding healthy fetal growth. Given that excessive GWG is strongly associated with macrosomia (41, 42), efforts to avoid excessive GWG are likely to simultaneously influence fetal fuel availability and birthweight in addition to potential direct influences of diet and dietary behavior on fetal tissue accretion (43). The higher prevalence of newborn SGA among women with high *pre-pregnancy* and *early pregnancy* diet score highlights that there is also a risky side of presumably healthy dietary behaviors, and that energy balance–related behaviors might compromise fetal fuel availability if taken too far. There was however no significant association between the diet scores and odds of SGA in multivariate analyses, indicating that other maternal characteristics associated with the diet scores might explain the increased prevalence of SGA in the unadjusted analysis in Table 2. Fortunately, the NFFD intervention did not result in increased prevalence of SGA in the intervention group compared to the control group (OR: 1.16, 95% CI: 0.68–2.00, *p* = 0.679) (2).

### Gestational diabetes mellitus

Surprisingly, the prevalence of GDM was substantially higher in the medium diet score category, whether assessed pre-pregnancy or in early pregnancy. This finding is difficult to explain and should be a target for further investigations in the NFFD dataset. The dietary differences between the diet score categories in Supplementary Table 2 give no clue as to why the medium diet score group should perform worse than the others. There may, however, be dietary characteristics specific to the medium diet score category that was not captured by the NFFD questionnaire. There could also be unmeasured confounding related to maternal risk status, for example, that women susceptible to overweight or obesity (and therefore at a higher risk of GDM) could be more conscious about their diet than women with lower risk, and therefore more likely to be categorized in the medium (or high) diet score category. A pre-pregnancy ‘Meats, snacks, and sweets’ pattern was associated with higher GDM risk, and a ‘Mediterranean-style’ pattern with lower risk after adjustment for socioeconomic, reproductive, and lifestyle factors in the Australian Longitudinal Study on Women’;s Health (44). Large population-based RCTs as well as RCTs targeting overweight and obese gravidas have demonstrated no effect of lifestyle interventions on GDM prevalence despite dietary improvement, increased physical activity, and in some of the studies, even reduced GWG (4, 5, 45). A recent systematic review on primary prevention studies of GDM through nutritional factors summarized that no conclusions can be drawn with regard to the best dietary interventions and that there is a strong need for additional research on this topic (46).

### Strengths and limitations

There are several strengths to the present study. The participation rate in the trial was high. All women provided dietary data upon inclusion in early pregnancy, and in retrospect, the same dietary data pertaining to the period before getting pregnant. Birth records and hospital charts were reviewed for all participants to confirm data on maternal weight gain and maternal and neonatal outcomes.

Only nulliparous women were included in the study. Nulliparity is associated with higher risk of pregnancy complications compared to multiparous pregnancies (16). Thus, heterogeneity and bias introduced by differential absolute risk of pregnancy complications between nulliparous and multiparous gravidas was avoided, as well as bias related to the high repeatability risk of preterm delivery and preeclampsia in subsequent pregnancies (16).

Sufficient variation in diet is necessary to detect true diet–disease associations in epidemiological studies (14). Randomized controlled diet interventions normally result in rather small mean improvements in diet or dietary behavior and are therefore not suited to assess dose–response relationships in diet–outcome associations (14). The NFFD diet score captured a continuum of combinations of healthy and less healthy dietary behaviors and thus much larger dietary variation than could be obtained in a RCT. Even though the numbers of preterm delivery, preeclampsia, and macrosomia cases were small in this study, we identified significant inverse associations even with these outcomes.

There are, however, also limitations that warrant discussion. Causality cannot be inferred from observational data. We adjusted for randomization assignment in all models and reran all models confined to the control group to avoid bias introduced by the intervention. There could, however, still be residual or unmeasured confounding from participating in a lifestyle intervention trial. The diet scores are likely to capture level of health consciousness in general, which could imply that variation in personal traits not captured by the sociodemographic variables could positively or negatively confound the associations.

The NFFD diet score and subscales have shown acceptable test–retest reproducibility (22) but have not been validated against other methods of operationalizing dietary behavior. Completing questionnaires about diet and frequency of intake challenges participants with complex tasks that increase the risk of misreporting (47). In addition, single-item questions about complex dietary behaviors may not fully capture a specific behavior. Recalling dietary behavior from before getting pregnant adds to the risk of misreporting and could lead to incorrect assignment of diet score values and a less reliable *pre-pregnancy* diet score in general. If random, misreporting would tend to bias the estimates toward null association. Misreporting or measurement error in the other covariates could not only lead to biased estimates but would also tend to bias estimates toward null association. We therefore assume that our estimates are conservative.

The NFFD diet score represents a crude operationalization of dietary behavior and equal weight is given to each dietary behavior regardless of their potential individual impact on the various outcomes. We did not investigate individual dietary behaviors in relation to the outcomes as our aim was to evaluate this constellation of dietary behaviors. It has previously been documented that single behaviors have less power as predictors of complex outcomes such as preterm delivery (48).

The NFFD diet score was primarily constructed to evaluate post-intervention effect of the diet intervention and reflected degree of adherence to the 10 dietary recommendations that were forwarded to the intervention group in the trial (22). It could be argued that it would be better to apply other criteria for scoring in each subscale rather than the statistically driven method of using the median in each subscale as cutoff. Similar methods have, however, been extensively used in evaluating associations between adherence to the Mediterranean diet and subsequent health outcomes (49).

The women participating in the NFFD study were predominantly white, European, highly educated, and nulliparous, and therefore not fully representative of the pregnant background population. This self-selection may have affected outcome prevalence but is less likely to have influenced diet-outcome associations (50).

## Concluding remarks

There are numerous reasons why pregnant women should be encouraged to achieve and maintain a healthy and nutritious diet during pregnancy. The relevance of pre-pregnancy diet for maternal and offspring antenatal and long-term health should, however, be explored as a means to securing maximum benefit of public health interventions. Our findings suggest that NFFD participants’ pre-pregnancy compliance with the dietary behaviors targeted in the NFFD trial was beneficially associated with risk of pregnancy and neonatal outcomes such as excessive GWG, preterm delivery, and newborn macrosomia. We did not observe substantial differences between associations with early pregnancy as opposed to pre-pregnancy dietary behavior, except for the lower odds of preeclampsia that was only observed with increasing *early pregnancy* diet score. Based on these findings, we speculate that the previously observed relationship between diet reported during pregnancy and pregnancy complications such as preterm delivery in observational studies could be a representation of a relationship that at least partly exists between maternal pre-pregnancy dietary behavior and the neonatal outcomes in question.

Prospectively collected high-quality dietary data from various time-points before conception and during pregnancy with the possibility of linkage to birth registry data would help identify important windows of opportunity for a healthy pregnancy and a nutritionally sound start to life. RCTs evaluating diet interventions before conception will need to be undertaken to establish causality.

## Supplementary Material

Pre-pregnancy and early pregnancy dietary behavior in relation to maternal and newborn health in the Norwegian Fit for Delivery study – a post hoc observational analysis
