# Supplementary material for: Pre-pregnancy and early pregnancy dietary behavior in relation to maternal and newborn health in the Norwegian Fit for Delivery study – a post hoc observational analysis
Source: Food Nutr Res. 2018 Aug 8;62:10.29219/fnr.v62.1273. doi: 10.29219/fnr.v62.1273 (PMC6085578; doi:10.29219/fnr.v62.1273)
Supplement: Pre-pregnancy and early pregnancy dietary behavior in relation to maternal and newborn health in the Norwegian Fit for Delivery study – a post hoc observational analysis [file FNR-62-1273-s001.pdf]

Table S1 Construction of the Norwegian Fit for Delivery Diet (NFFD) score from 10 subscales reflecting the dietary behaviors that were targeted in the NFFD diet intervention

Dietary recommendations	Related FFQ-question(s)	Response alternatives and coding	Calculations	Possible range of each subscale	Median = cut-off	Dietary behavior associated with scoring
1. Eat regular meals	How often do you eat<br>- breakfast<br>- lunch<br>- dinner<br>- evening meal/supper	Never = 0<br>Less than once a week = 0.5<br>Once a week = 1<br>Twice/week = 2<br>Three times/week = 3<br>Four times a week = 4<br>Five times/week = 5<br>Six times/week = 6<br>Every day = 7	Sum of answers to the four questions	0-28 meals a week	Pre-pregnancy: 23<br>Early pregnancy: 25	Pre-pregnancy:<br>< 24 meals/week = 0<br>≥ 24 meals/week = 1<br>Early pregnancy:<br>≤ 25 meals/week = 0<br>> 25 meals/week = 1
2. Drink water when thirsty	How often do you drink<br>- tap water<br>- bottled water<br>- carbonized water<br>- whole fat milk<br>- low fat milk<br>- skimmed milk<br>- juice<br>- fruit drinks or nectar<br>- sugar containing beverages<br>- artificially sweetened beverages<br>- alcohol containing beverages	Never = 0<br>Less than once a week = 0.5<br>Once a week = 1<br>Twice/week = 2<br>Three times/week = 3<br>Four times a week = 4<br>Five times/week = 5<br>Six times/week = 6<br>Every day = 7<br>Several times/day = 10	Sum of water intake events (frequency) divided by sum of all other drink intake events (frequency) X 100	0-100%	Pre-pregnancy: 44 %<br>Early pregnancy: 46%	Pre-pregnancy:<br>< 44% of drinking events is water=0<br>≥ 44% of drinking events is water = 1<br>Early pregnancy:<br>< 46% of drinking events is water = 0<br>≥ 46% of drinking events is water = 1
3. Eat vegetables with dinner every day	How often do you eat vegetables with dinner?	Never to daily (0-7)	No calculation	0-7	Pre-pregnancy: 4<br>Early pregnancy: 5	Pre-pregnancy:<br>≤ 4 times/week = 0<br>> 4 times/week = 1<br>Early pregnancy:<br>< 5 times/week = 0<br>≥ 5 times/week = 1

4. In between meals - choose fruits or vegetables	How often do you eat fruits or vegetables as an in-between meal?	Never to several times a day (0-10)	Frequency of eating fruits or vegetables in between meals	0-10	Pre-pregnancy: 3<br>Early pregnancy: 5	Pre-pregnancy:<br>< 3 times/week = 0<br>≥ 3 times/week = 1<br><br>Early pregnancy:<br>< 5 times/week = 0<br>≥ 5 times/week = 1
5. Eat sweets and snacks only when you really appreciate it	How often do you eat sweets or unhealthy foods without really appreciating it?	Never to several times a day (0-10)	No calculation	0-10	Pre-pregnancy: 0.5<br><br>Early pregnancy: 0.5	Pre-pregnancy and early pregnancy:<br>Never = 1<br>Sometimes = 0
6. Buy small portion sizes of unhealthy foods	Which size of the following three items do you usually buy?<br>- crisps<br>- chocolate<br>- soda	Small = 0<br>Big = 1	Sum of three answers	0-3	Pre-pregnancy: 1<br>Early pregnancy: 1	Pre-pregnancy and early pregnancy:<br>Buying small portion size of at least one of the three items = 1<br>Buying big size of all three items = 0
7. Limit your intake of added sugar	How often do you have<br>- sugar-sweetened fruit drinks<br>- soda<br>- cookies/crackers<br>- sweet breads<br>- cake/muffins<br>- sugar-containing - cereals<br>- sugar-containing - fruit yoghurt<br>- chocolate/sweets<br>How often do you add sugar to your food	Never to several times a day (0-10)	Sum of answers to the nine questions	0-90	Pre-pregnancy: 10.5<br>Early pregnancy: 10.5	Pre-pregnancy and early pregnancy:<br>< 10.5 (equivalent to eating food with added sugar once a day or less)= 1<br>≥ 10.5 (equivalent to eating food with added sugar more than once a day) = 0
8. Limit your intake of salt	How often do you eat<br>- salted crackers<br>- noodles<br>- crisps or other salted snacks<br>- hot dogs from kiosk/gas station	Never to several times a day (0-10)	Sum of answers to the seven questions	0-70	Pre-pregnancy: 6.5<br>Early pregnancy: 6.0	Pre-pregnancy:<br>Fast-foods, snacks or salting of food no more than 6.5 times a week = 1<br>Fast-foods, snacks or salting of foods more than 6.5 days a week = 0

	- fried potato chips from fast-food restaurants<br>- canned or freeze-dried food<br>How often do you add salt to your food?					Early pregnancy:<br>Fast-foods, snacks or salting of food no more than 6 times a week = 1<br>Fast-foods, snacks or salting of foods more than six days a week = 0
9. Do not eat beyond satiety	How often do you eat beyond satiety?	Never to several times a day (0-10)	No calculation	0-10	Pre-pregnancy:<br>1<br>Early pregnancy:<br>1	Pre-pregnancy and early pregnancy:<br>Less than once a week = 1<br>Once a week or more = 0
10. Read nutrition labels on foods before buying		Never=1<br>Sometimes=2<br>Usually=3<br>Always=4	No calculation	1-4	Pre-pregnancy:<br>2<br><br>Early pregnancy:<br>2	Pre-pregnancy and early pregnancy:<br>Never or seldom reading labels = 0<br>sometimes or often reading labels = 1
Total FFD-score			Sum of the ten dichotomized subscales	0-10		≤ 3 = low<br>4-5 = medium<br>≥ 6 = high

Table S2 Comparison of meal pattern and frequency of intake for selected foods across *pre-pregnancy* and *early pregnancy* diet score categories (n=591)

**Meal pattern and food intake (frequency per week)**	**Period for which diet was assessed**	**Low diet score**		**Medium diet score**		**High diet score**	
		Median	IQR	Median	IQR	Median	IQR
**Main meals per week**	Pre-pregnancy	21.0	17.0-23-0	23.0	21.0-25.0	25.0	23.0-28.0
	Early pregnancy	23.0	21.0-26.0	25.0	22.0-28.0	27.0	25.0-28.0
**Fruits (total)**	Pre-pregnancy	4.5	2.5-7.0	6.0	4.0-9.0	8.0	5.5-11.0
	Early pregnancy	7.0	4.5-9.0	9.0	6.5-11.0	10.0	8.0-13.0
**Vegetables (total)***	Pre-pregnancy	7.3	4.9-10.0	8.8	6.6-12.0	12.0	8.9-15.1
	Early pregnancy	8.1	5.7-10.8	10.0	7.5-12.6	12.8	9.8-15.8
**Vegetables with dinner**	Pre-pregnancy	3.0	2.0-4.0	4.0	3.0-6.0	6.0	5.0-7.0
	Early pregnancy	3.0	2.0-4.0	5.0	3.0-6.0	6.0	5.0-7.0
**Fruits and vegetables total**	Pre-pregnancy	11.8	8.1-16.6	15.3	11.7-19.8	19.6	16.0-24.8
	Early pregnancy	14.9	11.6-19.3	18.6	14.6-23.0	23.3	18.7-27.4
**Water (total)**	Pre-pregnancy	8.0	6.4-11.0	10.5	8.0-11.0	11.0	10.0-11.5
	Early pregnancy	10.5	7.5-11.0	10.5	8.0-11.0	11.0	10.0-12.0
**Milk (unsweetened)**	Pre-pregnancy	3.0	0.5-7.0	4.5	0.5-7.0	4.0	0.5-7.0
	Early pregnancy	5.0	2.0-7.4	5.0	1.0-7.0	5.5	0.5-7.0
**Sugar-sweetened beverages**	Pre-pregnancy	2.0	0.5-4.0	1.0	0.5-2.0	0.5	0.0-1.0
	Early pregnancy	2.0	0.5-4.0	1.0	0.5-2.0	0.5	0.0-1.0
**Artificially sweetened beverages**	Pre-pregnancy	3.0	0.5-6.0	1.0	0.5-3.0	0.5	0.0-2.0
	Early pregnancy	1.0	0.5-3.0	0.5	0.5-2.0	0.5	0.0-2.0
**Coffee**	Pre-pregnancy	2.0	0.0-7.0	5.0	0.0-10.0	7.0	0.5-10.0
	Early pregnancy	0.5	0.0-3.0	0.0	0.0-3.0	0.5	0.0-3.0
**Alcohol containing beverages**	Pre-pregnancy	0.5	0.5-1.0	0.5	0.5-1.0	0.5	0.5-0.5
	Early pregnancy	0.0	0.0-0.0	0.0	0.0-0.0	0.0	0.0-0.0
**Sweetened beverages combined****	Pre-pregnancy	10.0	6.9-12.1	7.0	4.5-9.8	5.0	2.5-8.0
	Early pregnancy	9.5	7.0-12.0	8.0	5.0-11.0	6.0	3.3-8.5
**Cakes and pastries**	Pre-pregnancy	2.0	1.5-3.5	1.5	1.5-2.0	1.5	1.0-2.0
	Early pregnancy	2.0	1.5-3.5	2.0	1.5-3.5	1.5	1.0-2.0

**Sweets and snacks**	Pre-pregnancy	5.0	4.0-8.0	4.0	2.5-5.0	3.0	2.0-4.0
	Early pregnancy	4.5	3.5-6.5	3.5	2.5-5.0	2.5	1.5-3.5

* frequency of eating vegetables, including vegetables used on sandwiches. Because of a supposedly small amount of vegetables on sandwiches, vegetables reported eaten on sandwiches were calculated as reported frequency divided by 4

**Sugar-sweetened and artificially sweetened beverages combined

Table S3a Sensitivity analysis of the associations between pre-pregnancy NFFD diet score and maternal and neonatal outcomes confined to the control group in the NFFD study (n=295)

Obstetrical outcomes	No. included in the analysis	No. of cases	Pre-pregnancy model 1<br>Crude			Pre-pregnancy model 2<br>Adjusted*		
			OR	**95% CI**	**p-value**	OR*	**95% CI**	**p-value**
**Adequacy of weight gain (term)[1]**								
**Excessive**	263	138	0.92	0.82-1.02	0.124	0.93	0.82-1.04	0.196
**Inadequate**	263	47	1.03	0.90-1.18	0.687	1.00	0.86-1.17	0.969
**Gestational diabetes[2]**								
**Elevated 2-hour glucose tolerance test (WHO criteria)**	287	23	1.02	0.84-1.23	0.843	1.03	0.84-1.26	0.794
**Preeclampsia[3]**								
**Preeclampsia total**	287	15	1.08	0.86-1.36	0.526	1.06	0.82-1.36	0.658
**Severe preeclampsia/HELLP/eclampsia[4]**	287	8	0.99	0.73-1.35	0.956	0.94	0.66-1.35	0.738
**Preterm delivery**								
**Prior to 37 weeks**	292	17	**0.77**	**0.61-0.97**	**0.029**	**0.79**	**0.61-1.01**	**0.060**
**Prior to 37 weeks (preeclampsia cases excluded)**	272	12	**0.64**	**0.47-0.86**	**0.003**	**0.66**	**0.47-0.92**	**0.015**
**Neonatal outcomes**								
**Birthweight > 4000 g (term)**	275	41	0.88	0.76-1.03	0.110	0.91	0.77-1.07	0.242
**Birthweight > 4500 g (term)**	275	5	0.87	0.58-1.30	0.488	0.84	0.53-1.31	0.438
**LGA >90th centile[5]**	292	11	0.85	0.64-1.12	0.234	0.86	0.64-1.16	0.335
**Birthweight < 2.5 kg (term)**	275	3	0.93	0.56-1.56	0.783	0.91	0.51-1.64	0.763
**SGA < 10th centile[5]**	292	26	1.04	0.87-1.25	0.643	1.03	0.85-1.24	0.806

LGA: large for gestational age; SGA: small for gestational age

*Multivariable associations between pre-pregnancy diet score and outcomes are expressed as odds ratios (OR) with 95 % confidence intervals (95% CI) and corresponding p-values. A p-value of ≤ 0.05 is considered significant. All models are adjusted for maternal age (continuous), educational attainment (≤ 12, 13-15, ≥16 years), marital status (cohabiting yes/no), income (4 categories), and pre-pregnancy BMI (continuous).

[1]Weight gain outside Institute of Medicine (IOM) 2009 recommendations (1)
[2] WHO 1999 criteria at gestational week 30: Elevated 2-h glucose ≥ 7.8 mmol/l (23)
[3] Based on guidelines adopted by the Norwegian Federation of Obstetricians and Gynecologists; an increase in blood pressure to at least ≥ 140 systolic or 90 mm Hg diastolic after 20th gestational week combined with proteinuria (protein excretion of at least 0.3 g/24 h or ≥ 1+ on dip-stick), both measured at least twice (24)
[4] Defined as preeclampsia diagnosed before 34 weeks of pregnancy and/or severity of symptoms, as documented in hospital charts. Cases of eclampsia and HELLP-syndrome were included.
[5] Birth weight percentile calculated according to offspring sex and gestational age, based on data from the Medical Birth Registry of Norway (MBRN)(26)

Table S3b Sensitivity analysis of the associations between early pregnancy NFFD diet score and maternal and neonatal outcomes confined to the control group in the NFFD study (n=591)

Obstetrical outcomes	No. included in the analysis	No. of cases	Early pregnancy model 1 Crude			Early pregnancy model 2 Adjusted*			Early pregnancy model 3 Adjusted**		
			OR	**95% CI**	**p-value**	OR*	**95% CI**	**p-value**	OR**	**95% CI**	**p-value**
**Adequacy of weight gain (term)[1]**											
**Excessive (yes/no)**	263	138	**0.88**	**0.78-1.00**	**0.043**	**0.86**	**0.75-0.98**	**0.020**	**0.80**	**0.69-0.93**	**0.003**
**Inadequate (yes/no)**	263	47	1.08	0.92-1.26	0.345	1.11	0.94-1.30	0.240	1.14	0.95-1.36	0.156
**Gestational diabetes[2]**											
**Elevated 2-hour glucose tolerance test (WHO criteria)**	287	23	0.99	0.81-1.22	0.943	0.97	0.78-1.20	0.762	1.01	0.77-1.32	0.945
**Preeclampsia[3]**											
**Preeclampsia total**	287	15	0.99	0.77-1.27	0.935	0.95	0.73-1.24	0.714	0.91	0.69-1.20	0.503
**Severe preeclampsia/HELLP/eclampsia[4]**	287	8	0.88	0.62-1.25	0.485	0.83	0.57-1.21	0.333	0.83	0.57-1.21	0.333
**Preterm delivery**											
**Prior to 37 weeks**	292	17	**0.68**	**0.52-0.89**	**0.004**	**0.67**	**0.50-0.89**	**0.007**	**0.73**	**0.54-0.99**	**0.040**
**Prior to 37 weeks (preeclampsia cases excluded)**	272	12	**0.54**	**0.38-0.77**	**0.001**	**0.51**	**0.34-0.77**	**0.001**	**0.53**	**0.33-0.86**	**0.010**
**Neonatal outcomes**											
**Birthweight > 4000 g (term)**	275	41	**0.83**	**0.70-0.98**	**0.028**	0.84	0.69-1.01	0.063	0.83	0.68-1.02	0.081
**Birthweight > 4500 g (term)**	275	5	**0.57**	**0.35-0.95**	**0.030**	**0.52**	**0.30-0.88**	**0.015**	0.60	0.33-1.10	0.097
**LGA >90th centile[5]**	292	11	0.78	0.57-1.07	0.121	0.77	0.55-1.08	0.127	0.76	0.50-1.17	0.215
**Birthweight < 2.5 kg (term)**	275	3	0.97	0.56-1.3	0.969	1.04	0.53-2.04	0.917	0.98	0.51-1.89	0.948
**SGA < 10th centile[5]**	292	26	1.11	0.91-1.35	0.292	1.11	0.91-1.37	0.308	1.12	0.90-1.41	0.311

LGA: large for gestational age; SGA: small for gestational age

*Multivariable associations between early pregnancy diet score and the outcomes are expressed as odds ratios (OR) with 95 % confidence intervals (95% CI) and corresponding p-values. A p-value of ≤ 0.05 is considered significant. Model 2 is adjusted for maternal age (continuous), educational attainment (≤ 12, 13-15, ≥16 years), marital status (married/cohabiting yes/no), family income (4 categories), current smoking (yes/no) and pre-pregnancy BMI (continuous)
**Model 3 is additionally adjusted for early pregnancy physical activity level

[1]Weight gain outside Institute of Medicine (IOM) 2009 recommendations (1)

[2] WHO 1999 criteria at gestational week 30: Elevated 2-h glucose ≥ 7.8 mmol/l (23)

[3] Based on guidelines adopted by the Norwegian Federation of Obstetricians and Gynecologists; an increase in blood pressure to at least ≥ 140 systolic or 90 mm Hg diastolic after 20th gestational week combined with proteinuria (protein excretion of at least 0.3 g/24 h or ≥ 1+ on dip-stick), both measured at least twice (24)
[4] Defined as preeclampsia diagnosed before 34 weeks of pregnancy and/or severity of symptoms, as documented in hospital charts. Cases of eclampsia and HELLP-syndrome were included.
[5] Birth weight percentile calculated according to offspring sex and gestational age, based on data from the Medical Birth Registry of Norway (MBRN) (26)
